# Formation of Future Educators’ Professional Training for Introducing Social Experience by Means of Innovative Technologies of Education to Senior Preschoolers

**DOI:** 10.3390/bs10020042

**Published:** 2020-01-25

**Authors:** Marharyta Shkabarina, Liliya Melnychuk, Vadim Koval, Svitlana Stupnitska

**Affiliations:** 1 Department of Ukrainian Language and Literature, International University of Economics and Humanities named after Academician Stepan Demianchuk, st. acad. S.Demianchuk, 4, 33027 Rivne, Ukraine; 2 Department of Pedagogics, International University of Economics and Humanities named after Academician Stepan Demianchuk, st. acad. S.Demianchuk, 4, 33027 Rivne, Ukraine; lilya1154@ukr.net; 3 Department of Human Health and Physical Therapy, International University of Economics and Humanities named after Academician Stepan Demianchuk, st. acad. S.Demianchuk, 4, 33027 Rivne, Ukraine; lara.kv@ukr.net (V.K.); svitstup@ukr.net (S.S.)

**Keywords:** professional training, innovative teaching technologies, social experience

## Abstract

The purpose of the paper is to reveal the peculiarities of the introduction of innovative education technologies in the process of training future pre-school teachers in the field of the socialization of preschoolers, and to verify their effectiveness by experimental means. During the research, the methods of analysis and synthesis of philosophical, psychological and pedagogical sources in the field of professional training of the future teacher were used: modeling and designing, to determine the theoretical and methodological foundations of the research and development of innovative technology for the training of future teachers to familiarize preschoolers with social environments; studying and generalizing the current state of the professional training of future teachers of pre-school institutions to familiarize preschoolers with social reality; comparison and classification to determine the essential characteristics, criteria and levels of readiness of future pre-school teachers for the socialization of preschoolers; pedagogical experiment with qualitative and quantitative analysis of results, in particular, the statistical criterion of K. Pearson (x^2^). The results of the diagnosis led to the need to implement innovative educational technologies into the process of the professional training of future teachers of pre-school educational institutions in three stages: diagnostic and propaedeutic, motivational, activity–creative.

## 1. Introduction

Modern democratic processes of reformation and modernization that are currently taking place in the higher education system are objectively aimed at its further progressive development, as well as providing for the needs of the highly qualified specialists. This implies corresponding changes in the professional and pedagogical training of teachers who are to work with preschool children of different age-groups.

According to Rogalskaya [1], making children aware of the different phenomena of social life lies at the root of mastering the native language and developing a national consciousness. The child’s introduction into the environment ensures the natural development of their character in the process of perceiving reality, and brings the content of education closer to the natural, subjective, and social environment. Therefore, the problem of developing the child’s interest in social reality and the phenomena of social life at this favorable stage of its childhood is becoming more urgent.

The future teacher’s readiness to familiarize preschoolers with social reality should come as the result of thorough professional training, and the formation of this readiness incorporates the formation of a system of particular motives, interests, attitudes, personality traits, knowledge, and practical experience, which, when activated, can provide the possibility for them to effectively perform their professional functions. The challenges of training future teachers are related to the processes of improving preschool education in general: finding new content, forms, and methods for it, as well as restructuring this system on the basis of personally oriented education, which entails the introduction of innovative pedagogical technologies.

What are innovative pedagogical technologies? According to Klarin [2], innovative pedagogical technology is the purposeful, systematic and consistent implementation of original, innovative methods, techniques, pedagogical actions and tools that cover the whole educational process, from the definition of its purpose to the expected results in practice.

Scientists Ahmetova et al. [3], Kopf et al. [4] and Smith [5] argue that the main features and benefits of innovation education are:

- The orientation of the development of each student’s personality, the formation of his readiness for real life and professional activity, and the development of creative thinking, critical analysis of the surrounding world and ourselves in it;
- The formation of the readiness of the student for constant mastering of new kinds of activities and communication;
- A high level of activity of students, where the teacher acts as a teacher–manager and director of training, the student acts as a subject of activity with the teacher, and his personal development acts as one of the main educational goals;
- Encouragement and support of students’ initiatives, a gradual and purposeful transition from education and training to the formation of the ability to self-educate and self-train;
- The participation of each student in the process of defining goals, tasks, and decision-making;
- In the process of learning, the essential forces of each individual are revealed and involved, mobilizing their goals, abilities, and giftedness;
- The creative, research and productive tasks which determine the essence and motives of the choice of educational reproductive tasks, are considered as the most significant;
- Interdependence and self-control prevail within the common values and content of the whole group;
- A new type of organization of the student’s motivational sphere is formed, where the motives of self-actualization, co-creation and self-knowledge influence the general creativity of the student and contribute to the creation of a new position of the individual.

According to Cherkasov [6] and Ronald [7], the following technologies are referred to as innovative technologies: technology of situational learning (case-method); gaming technology; design technology; technology of problem learning; information and communication technologies (ICT); technology of solving inventive tasks, portfolio.

The various aspects of the professional training of students of the specialty 012 “Preschool education” were the subject of research in modern scientific papers, such as those by Saiko [8] and Dubasenyuk [9]. Scientists are considering the problems of training teachers in higher education institutions for future social and pedagogical activities, alongside with the training of preschool teachers in the sphere of socializing preschool children. At the same time, special scientific interest should be paid to the pedagogical strategy for the development of the innovative personal orientation of preschool teachers, who can envision new approaches to children, and the specifics of their socialization in modern society, and are able to provide the required social and pedagogical support for acquainting preschoolers with various social issues.

Modern science can boast of considerable scientific and pedagogical legacy in the problem of preparing future preschool teachers to familiarize children with social reality, but its various aspects are still relevant, for the analysis of the current practices of educating preschool children shows that a teacher with an undeveloped social consciousness, system of social values, and potential for innovation, as well as an insufficient level of professional and pedagogical culture, cannot fully involve their educatees in the process of their socialization. This proves the need to make adjustments to the content and methods of preschool teachers’ professional training in the sphere of familiarizing preschoolers with social reality through innovative technologies.

However, the problem of ensuring the professional readiness of future preschool teachers to familiarize preschoolers with social reality by means of innovative educational technologies has not always been easy to research.

## 2. Materials and Methods

The main research questions are:

1. Is the strategy of innovative educational technologies effective in the process of forming the professional readiness of future teachers of preschool institutions to familiarize preschoolers with social reality?

2. What types of innovative pedagogical technologies are the most effective in future preschool teachers training?

3. Will the introduction of innovative educational technologies in the process of the professional training of students of the specialty 012 “Preschool education” influence the growth of qualitative indicators of readiness of future teachers of pre-school institutions to familiarize senior preschool children with social reality?

The purpose of the study is to reveal the peculiarities of implementing innovative educational technologies into the process of training future teachers of preschool institutions to acquaint preschoolers with social reality in classes on educational disciplines—”Method of familiarizing preschoolers with social environments”, “Theory and methods of familiarizing preschool children with ethnography”, “Theory and methodology of cooperation with families”, “Innovative pedagogical technologies in pre-school education—and to check the effectiveness of the experiment. 

The students of specialty 012 “Preschool education”, of 19–21 years of age who study two, three, and four courses, were the subjects of this study. In total, 400 students were involved in the experiment, of which 195 students were in the experimental group and 205 were in the control group. The research was conducted on the basis of the International University of Economics and Humanities, named after Academician Stepan Demianchuk, Rivne State Humanitarian University, Lesia Ukrainka Eastern European National University, and Kremenets Regional Taras Shevchenko Humanitarian and Pedagogical Academy. All participants gave their informed consent for inclusion before participating in the study. The Ethical Committee of the Primary and Pre-school Education of International university of economics and humanities, named after academician Stepan Demianchuk, and furthermore, the study was conducted in accordance with the Declaration of Helsinki.

At the first stage of the pedagogical experiment, a diagnosis of the professional readiness levels of future teachers to familiarize senior preschoolers with social reality, in accordance with developed criteria and indicators, was performed. In the professional training of students of the experimental group, a complex of innovative educational technologies was introduced that, hypothetically, would influence the growth of qualitative and quantitative indicators of the readiness of future preschool teachers to familiarize preschoolers with social reality.

We consider the formation of the future teachers’ professional readiness in the context of acquainting preschoolers with the social environment to be a very complex system; therefore, we have identified the criteria that reflect a certain state of its development as a systematic factor in the procedural aspect of the professional preparation, that is, in the process of studying at the university, with one clear result—teach students to perform the independent professional activities related to the development of the preschoolers’ socialization.

The following aspects were considered when substantiating the criteria and indicators of future educators’ readiness to familiarize preschoolers with the social environment:

- The peculiarities of professional and pedagogical training, as well as the specifics of the professional activities of future preschool educators in the context of acquainting preschool children with social reality;
- The essence of professional activity as a complex of the defined personality traits which should be formed in the process of university preparation and can provide a high level of readiness to familiarize preschoolers with the social environment;
- The structure, interconnections, and interdependence of the structural components of professional training in the context of the identified problem.

Based on the results of the consulting stage of the research, three levels of future preschool teachers’ readiness to familiarize preschoolers with the social environment were determined, namely: creative (high), medium (reproductive), and low (adaptive).

The assessment of the level of the future specialists of preschool education’s readiness to familiarize children with the social environment was carried out according to three criteria: motivational-targeted, content-procedural, and activity criterion. For the diagnosis of the motivational-targeted criterion, the method Ilyin—“Motivation of studying at a university” [10]—was chosen. Diagnosis of the content-procedural criterion was performed on the basis of the examination session. Diagnosis of the formation of the indicated readiness by activity criterion was carried out with the help of the questionnaire “Self-assessment of professional skills” [11].

Diagnosis of the professional readiness of future eteachers to familiarize preschoolers with the social environment was made before and after the experiment. For our research it is important to compare the results of both students’ experimental and control groups’ studied criteria. For this purpose, we chose the nonparametric criterion X² (Pearson’s chi-squared test) [12], which allows us to compare the distribution of the objects of two sets by a condition of a certain quality on the basis of measurements on the scale of the names of that quality in two independent samples. According to the results (Table 1), the number of future teachers before the experiment with the adaptive level of professional readiness to familiarize preschoolers with the social environment was 20% in the control group and 19.5% in the experimental group. There were 59.1% of students with a reproductive level in the control group and 59% in the experimental group, and 20.9% of students had a students with creative level in the control group and 21.5% did in the experimental group. The calculation of the Pearson chi-squared test according to the final data was X² = 0.027. This is less than the critical value of 5.991 (p = 0.95)—that is, these two groups are similar in terms of their level of professional readiness to familiarize preschoolers with the social environment. Analysis of the initial assessment demonstrated that future preschool teachers show medium and low quantitative indicators of their level of readiness to familiarize children with the social environment.

Therefore, there arose a need to introduce innovative technologies aimed at the increase in this level, as well as the need to check its effectiveness during the formative experiment.

The innovative technology for training future preschool teachers in the sphere of preparing children to getting acquainted with the social environment envisages the realization of the following pedagogical conditions: motivating the students to master professional activities in the context of preschoolers’ socialization; mastering students’ knowledge in the sphere of the preschoolers’ socialization and acquiring practical skills for its application; introducing a set of both traditional and innovative forms and methods into a holistic system of future preschool teachers’ professional training.

The implementation of innovative educational technologies is completed in three stages: (1) diagnostic and propaedeutic; (2) motivational; (3) with activities and creativity.

The purpose of the first stage—a combination of diagnostic and propaedeutic technologies (two years of studying)—provides the active involvement of students in the system “man-profession” at the stage of training, stimulation of professional motivation.

The conditions of the successful implementation of the goal of the diagnostic and propaedeutic stage are provided: the humanistic orientation of the student’s personality, where his professional outlook and professional self-consciousness are formed; the development of a constant need for self-perfection and self-development on a reflexive basis has been made; there has been development of professional dignity and professional duty.

This contributed to the formation of a stable positive motivation to master professional skills and broaden the ideas of students about the orientation of the individual, the components of professional activity and its importance in the daily activities of the educator.

The second goal—the motivational stage (III course)—was the formation of professional values for future preschool teachers, and encouraged positive attitudes toward future activities in a pre-school educational institution.

Conditions for the successful implementation of the diagnostic-propaedeutic stage are as follows: the system of professional values is formed in future preschool teachers, as well as motivation for positive attitudes towards future activity in the pre-school educational institution.

At this stage, one of the most essential aspects to consider was the motivation of future preschool teachers to familiarize preschoolers with the social environment. Future preschool teachers have become acquainted with the qualities that a preschool teacher has to possess, as well as the importance and the responsibility of this occupation. The positive aspects of future educators’ preparation to introduce preschool children to the social environment are highlighted, and the students are shown the importance of this process.

The process of creating positive motivational support for the educational process was implemented during practical classes, where students determined the necessary complex of professional qualities, personal traits, and pedagogical abilities that contribute to the formation of the professional competences of a future preschool teacher. Positive motivational support for the educational process in the higher education institution has increased the students’ cognitive activity, their interest in their future profession, and intensified the need to develop professional competence in the context of the preschoolers’ socialization.

This work contributed to the development of students’ perceptions of the impact of success and failure on professional growth, well-being, relationships with other subjects of the pedagogical process, and developed their need for successful activities in the future, as well as the desire to master professional competencies.

The leading method of future preschool teachers’ preparation for the aforementioned type of work at the motivational stage consisted of the exercises for the identification and correction of students’ professional motivations, focusing on self-development and self-improvement on a constant reflexive basis, understanding the value of the chosen pedagogical occupation, and self-identification as a preschool teacher.

The final result of the introduction of the motivation stage for the training for teachers to familiarize preschoolers with social reality was the positive attitude of future educators towards the identified activity and awareness of its importance; the formation of professional values, acquaintance with the professionogram of the teachers of preschool educational institutions; and the preparedness of students for active, creative (individual and group) activities.

The purpose of the third—activity–creative stage (IV course)—was determined as the active involvement of students in the formation of competencies in the context of preschoolers’ socialization in the educational process during the study of professional disciplines and the acquisition of knowledge about the essence, content and methods of familiarizing preschoolers with social reality.

The process of the mastering the system of knowledge of acquainting preschool children with the social environment, and acquisition of the skills for its further practical application, happened with the help of the following factors: assisting students in the implementation of the required knowledge and the application of these skills in the course of the pedagogical internships in preschool education institutions; providing pedagogical and psychological support for preschoolers; assistance in solving conflict situations in the children’s team; working with the preschoolers on organizational day-to-day activities; giving individual lessons with the purpose of the preschoolers’ socialization; organizing different age-group activities for studying the social environment.

The experimental work on the preparation of future teachers for familiarizing preschoolers with the social environment at the activity–creative stage comprised: studying materials regarding normative educational disciplines, an optional course “Methodology of acquainting preschool children with the social environment”, pedagogical internship in the institutions of preschool education; and the forms of work (lectures (lectures–conferences, problematic lectures, lectures–discussions, lectures with analysis of specific situations), seminars, independent studying activities, independent research work, consultations, seminars, trainings, discussions).

The stated goal is realized by stabilizing and correcting the formed psychological and pedagogical competencies of students of the specialty 012 “Preschool education”, the formation of an individual style of professional activity in the context of preschoolers’ socialization, the formation of the ability to self-assess their acquired experience, the consolidation of the skills and abilities of independent and research work. The conditions for the realization of the activity–creative stage goal are provided: informational and methodical provision for the training of future pre-school educators through a combination of traditional and innovative forms of training, with the integration of the content found in normative educational disciplines (“Theory and method of familiarizing preschool children with ethnography”, “Theory and method of cooperation with families”, “Innovative pedagogical technologies in pre-school education”) and the curriculum with the variable components of the program (students’ choice), “Method for familiarizing preschoolers with social environments”; professionally oriented technologies (contextual learning with a combination of innovative technologies, project activity, exchange of pedagogical experience, pedagogical portfolio); interactive technologies aimed at creating a subject–subjective relationship between teacher and student (cooperative learning, pair training, frontal education, learning in the game form, learning in the discussion form) are presented.

Students’ professional experience, gained in the process of solving pedagogical situations, creative and practical tasks, as well as passing pedagogical internships, helped synthesize the results of previous theoretical and practical training. The conditions created during the “Methodology of acquainting preschool children with the social environment” classes were actually evaluated by students as paramount for the formation of their professional potential and professional competences in the context of the socialization of preschoolers.

The consequences of implementing the activity–creative (main) stage were the possession of knowledge about the essence, content, tasks and methods of familiarizing preschoolers with social environments and the innovative technologies of teaching preschoolers, raising the level of development of personal and professional qualities.

In all stages of implementing the technology required for the professional readiness of future educators to familiarize senior preschool children with social reality, the opportunities of information and communication technologies of learning were used, namely, computerized sets for the development of multimedia training courses (Macromedia AuthorWare, Adobe Captivate, Articulate Studio, iSpring Suite, eLearinng Office 3000), video tutorials (Jing, Webineria, Wink, Camtasia Studio, WindowsMediaEncoder), test tasks (MyTest, OpenTEST2, Assistant2).

## 3. Results

As a result of the final stage of the work, in order to analyze the dynamics of the changes that have occurred through the introduction of experimental technology, a final assessment was made according to three criteria: motivational–targeted, content–procedural, and activity criterion.

Based on the results of the research, three levels of future preschool educators’ readiness to familiarize preschoolers with the social environment were determined, namely: high (creative), medium (reproductive), and low (adaptive).

The creative level of readiness to familiarize preschoolers with the social environment is common for students who are motivated for pedagogical activities. They are characterized by a high level of motivation, which provides professional interest in the process of familiarizing preschoolers with social reality.

The students of this level are familiar with the key pedagogical categories, and the essence and content of professional methods; they express considerable interest and a desire to improve their professional and pedagogical competence and strive for self-development and self-improvement. They also possess advanced communication skills and know how to create appropriate educational socio-cultural micro-environments. The students have a capacity for introspection, can relate themselves and their actions to the behavior of others. Under various circumstances, this can contribute to the development of reflexivity, an important component of their future professional activities.

The reproductive level of readiness for acquainting preschoolers with the social environment is characteristic of the students who have partially acquired professional knowledge and skills and do not always feel a subjective need for their replenishment and deepening, as evidenced by the average level of their motivation for success and professional motivation. The future teachers understand the essence of the “social environment” concept but are only partially aware of the purpose of and tasks required for preschoolers’ socialization. The lack of continuous motivation to improve the results of professional training causes these students to demonstrate superficial knowledge and skills in the professional methodology, as well as low awareness of the importance of communication patterns, the need for reflexivity, and the ability to empathize. The medium level of communicative abilities’ formation does not allow them to easily establish professional–pedagogical interactions with their future pre-school children; such students have difficulty reflecting on their activities and are not sufficiently engaged in self-development.

The adaptive level of readiness to familiarize preschoolers with social reality is characteristic of students who reproduce knowledge mechanically, and are not interested in improving their skills and ability to work with future pre-school children in the context of their socialization, which indicates a complete lack of motivation to perform pedagogical activities. The future professionals equate the concepts of “social environment” and “natural environment”, and do not realize the goals and objectives of preschoolers’ socialization. The students of this level have poorly developed communication skills, alongside considerable difficulties in self-analysis and self-assessment. These future specialists in preschool education are not aware of the importance of mastering the various components of their professional activity. Such lack of interest in the development of professional skills and abilities has led to the inability to develop the preschoolers’ knowledge of social reality phenomena and engage into social relationships. Because of this, the children are not always capable of establishing friendly relations and interactions with both their peers and the other people surrounding them.

After the experiment was finished, we managed to observe positive dynamics reflected in the increase in the professional readiness level among the Experimental Group students, while in the Control Group these changes were less significant. Comparing the data before and after the experimental work (Table 1), we note that the students in the experimental group at the adaptive level decreased by 11.2%, the number of students at the reproductive decreased by 1.1%, and the number of future educators at the creative level increased by 12.3%. The results of the distribution of students in the control group indicate the following changes: the number of students at the adaptive level decreased by 2.4%, the number of students at the reproductive level increased by 0.9%, and the students at the creative level increased by 1.5%. Comparing the data in the control group before and after the experiment, we obtained values of Х²Эмп = 0.442, less than the critical value (Х² = 5.991). Thus, 0.442 < 5.991 for p = 0.95 (X^2^Emp <X^2^). Therefore, the level of professional readiness to familiarize preschool students with the social environment in the control group did not change after the experiment.

Comparing the data in the experimental group before and after the experiment, we obtained the value of X² = 14.314, which is more critical, which, for significance p = 0.95, is X^2^ = 5.991. Thus, 14.314> 5.991 (X^2^Emp> X^2^). Thus, the level of professional readiness to familiarize preschoolers with the social environment in the experimental group increased after the experiment. The results of the controlling assessment have shown that the students from the EG, where we used the innovative educational methodology, have demonstrated some statistically significant shifts towards an increase in the future preschool teachers’ professional readiness to familiarize older preschoolers with social reality.

## 4. Discussion and Conclusions

Answering the Research Questions:

1. The strategy of innovative educational technologies is effective in the process of increasing the professional readiness of future teachers of preschool institutions to familiarize preschoolers with social reality. The professional readiness of future preschool teachers to introduce the social reality to preschoolers is an integrated form of personality aimed at the effective solving of educational tasks, with the help of a system of psychological, pedagogical and methodological competencies, motives and social and moral values. The qualities and abilities of the teacher are necessary for the effective performance of all types of social and pedagogical activities. Teachers were in the process of studying at a higher education institution aiming to develop independent professional activities related to the development of preschool socialization;

2. The most effective techniques in future educator training are the following: innovative teaching technologies, such as situational learning technology (case-method), gaming, design technology; technology of problem learning; information and communication technologies, portfolio;

3. The effectiveness of the technology of training future preschool teachers to introduce the social environment to preschoolers by means of innovative technologies is confirmed by positive changes in the indicators of professional readiness after the formative stage of the experiment. 

## Figures and Tables

**Table 1:** Dynamics of future educators’ professional training of introducing social experience.

Stages	Control Group (CG)				Experimental Group (EG)			
	Before the Experiment (n = 205)		After the Experiment (n = 205)		Before the Experiment (m = 195)		After the Experiment (m = 195)	
	f	%	f	%	f	%	f	%
Adaptive	41	20	36	17.6	38	19.5	16	8.3
Reproductive	121	59.1	123	60	115	59	113	57.9
Creative	43	20.9	46	22.4	42	21.5	66	33.8
